# Expression of costimulatory molecules in human neuroblastoma. Evidence that CD40+ neuroblastoma cells undergo apoptosis following interaction with CD40L

**DOI:** 10.1038/sj.bjc.6600951

**Published:** 2003-05-13

**Authors:** I Airoldi, S Lualdi, S Bruno, L Raffaghello, M Occhino, C Gambini, V Pistoia, M V Corrias

**Affiliations:** 1 Laboratory of Oncology, G Gaslini Institute, Largo G Gaslini, 5, 16148 Genova, Italy; 2 Department of Experimental Medicine, Section of Human Anatomy, University of Genova, Genova, Italy; 3 Service of Pathology, G Gaslini Institute, 16148 Genova, Italy

**Keywords:** costimulatory molecules, human neuroblastoma, CD40, apoptosis, caspase-8

## Abstract

Tumour cells display low to absent expression of costimulatory molecules. Here, we have investigated the expression of costimulatory molecules (CD40, CD80, CD86, PD-1L, B7H2, OX40L and 4-1BBL) in human neuroblastoma (NB) cells, since virtually no information is available on this issue. Both established NB cell lines and primary tumours were tested by RT–PCR and flow cytometry. Neuroblastoma cell lines expressed the transcripts of all costimulatory molecule genes, but not the corresponding proteins. Culture of NB cell lines with human recombinant (r)IFN-*γ* induced surface expression of CD40 in half of them. Primary NB cells showed CD40, CD80, CD86, OX40L, 4-1BBL, but not PD-1L and B7H2, mRNA expression. Surface CD40 was consistently detected on primary NB cells by flow cytometry. Interferon-*γ* gene-transfected NB cells expressed constitutively surface CD40 and were induced into apoptosis by incubation with rCD40L through a caspase-8-dependent mechanism. CD40 may represent a novel therapeutic target in NB.

Costimulatory molecules play an indispensable role in the induction and maintenance of T-cell activation. Costimulatory signals promote T-cell proliferation, cytokine production and effector functions (Chambers *et al*, 2001). T-cell activation through T-cell receptor (TCR) and cluster designation 28 (CD28) induces CD8+ T lymphocytes to acquire cytolytic functions and CD4+ cells to differentiate into T Helper 1 (TH1) or TH2 cells (Lenschow *et al*, 1996). Some costimulatory molecules, such as CD40, CD80 and CD86, are constitutively expressed by professional antigen-presenting cells (APCs), and rapidly upregulated in the course of antigen presentation to T cells (Chambers *et al*, 2001). The binding of CD80 and CD86 on APC to CD28 on T cells is followed by the upregulation of additional costimulatory molecules on the latter cells, that amplify (e.g. inducible costimulator (ICOS) and 4-1BB; Akiba *et al*, 1999; Wang *et al*, 2000; Cannons *et al*, 2001; Dong *et al*, 2001) or downregulate (e.g. programmed death 1 (PD-1); Freeman *et al*, 2000) T-cell activation upon binding to their receptors on APC. On the other hand, interaction between CD80 and CD86 on APC with CTLA-4 on T cells delivers inhibitory signals to the latter cells that dampen ongoing immune responses (Chambers, 2001).

T-cell interaction with nonprofessional APC-presenting antigenic peptides in the context of HLA-class I or II molecules, but in the absence of costimulatory molecules, leads to T-cell anergy or apoptosis (Chambers, 2001).

Most tumour cells are poor APC because of the low or absent expression of costimulatory molecules (Hurwitz *et al*, 2000), but enforced expression of some of these (e.g. CD80, B7H2 or 4-1BBL) by gene transfer has resulted in enhanced antitumour responses and tumour rejection in different animal models (Wu *et al*, 1995; Weinberg *et al*, 2000; Liu *et al*, 2001).

Neuroblastoma (NB) is the most common solid tumour in young children and carries a poor prognosis in more than half of the cases, because of its frequent presentation as metastatic disease at diagnosis (Brodeur *et al*, 1988). High-dose chemotherapy followed by rescue with autologous haematopoietic stem cells has not improved significantly the outcome of advanced-stage NB patients.

So far, two groups have reported that CD80 or CD86 gene transfer in murine NB cells decreases their tumorigenicity (Katsanis *et al*, 1995; Enomoto *et al*, 1997), whereas virtually no data are available on the expression of costimulatory molecules in human NB cells (Uçar *et al*, 1995). In this perspective, we have investigated the expression of the CD40, CD80, CD86, PD-1L, B7H2, OX40L and 4-1BBL costimulatory molecules in 10 human NB cell lines, as well as in primary tumour cells isolated from NB patients. Here, we show that incubation of surface CD40+ NB cells with CD40 ligand (L) triggers their apoptosis through a caspase-8-dependent mechanism.

## RESULTS

### Expression of CD40, CD80, CD86, PD-1L, B7H2, OX40L and 4-1BBL in NB cell lines

A total of 10 human NB cell lines were tested for the expression of costimulatory molecules by RT–PCR and, when reagents were available, by flow cytometry.

**Figure 1:**
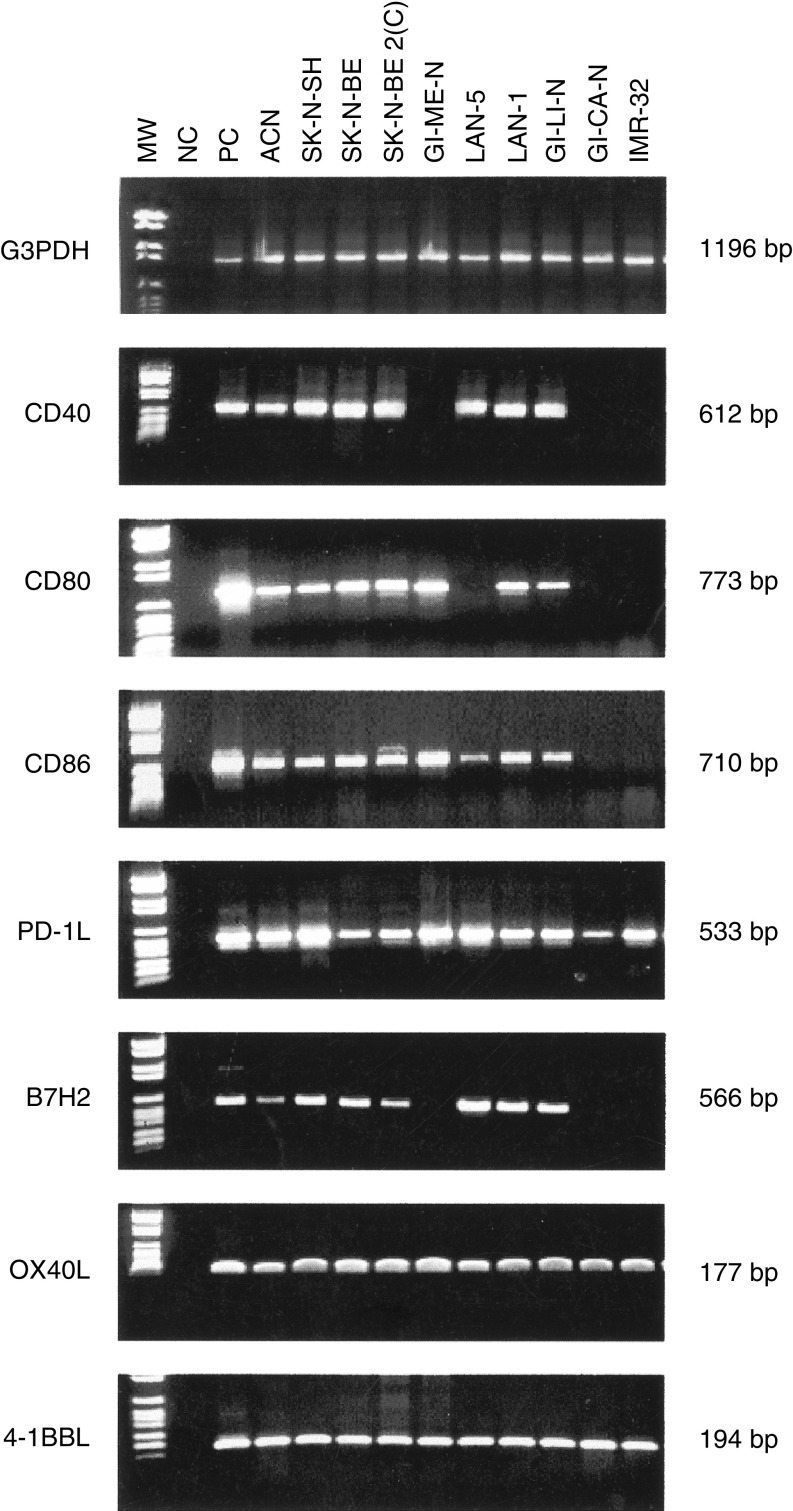
Costimulatory molecule gene expression in human NB cell lines, as assessed by RT–PCR. From left to right: MW=molecular weight markers; NC=negative control, represented by water in the place of cDNA; PC=positive control, represented by normal peripheral blood MNC stimulated with phytohaemagglutinin for 6 h; lanes from 4 to 13, 10 NB cell lines are shown. The first upper panel shows the amplification product of the G3PDH housekeeping gene tested as control. On the right side of each panel, the expected MW of the amplification products are shown.

CD40 and B7H2 mRNA were detected in all the cell lines but GI-ME-N, GI-CA-N and IMR-32. CD80 and CD86 transcripts were present in the majority of NB cell lines; exceptions were LAN-5, that did not express the CD80 gene, as well as IMR-32 and GI-CA-N cells, that did not show CD80 or CD86 transcripts (Figure 1

). PD-1L, OX40L and 4-1BBL mRNA were found in all the cell lines (Figure 1). The specificity of the amplified bands was confirmed by sequencing.

In subsequent experiments, surface expression of CD40, CD80, CD86, 4-1BBL and OX40L on the 10 NB cell lines was investigated by flow cytometry. Cells were stained with CD40, CD80 and CD86 monoclonal antibodies (mAbs), as well as with the rhOX40:Fc and rh4-1BB:Fc fusion proteins. No costimulatory molecule was detected on the surface of any cell line, with the exception of OX40L that was expressed by GI-CA-N and IMR-32 cells only (not shown).

### Recombinant (r) interferon-*γ* (IFN-*γ*) treated NB cell lines express surface CD40

In further experiments, NB cell lines were incubated with human rIFN-*γ* for 72 h, since this cytokine upregulates the expression of various costimulatory molecules (Aicher *et al*, 2000; Wagner *et al*, 2002). Thereafter, costimulatory molecule gene expression was investigated by RT–PCR and flow cytometry.

**Figure 2:**
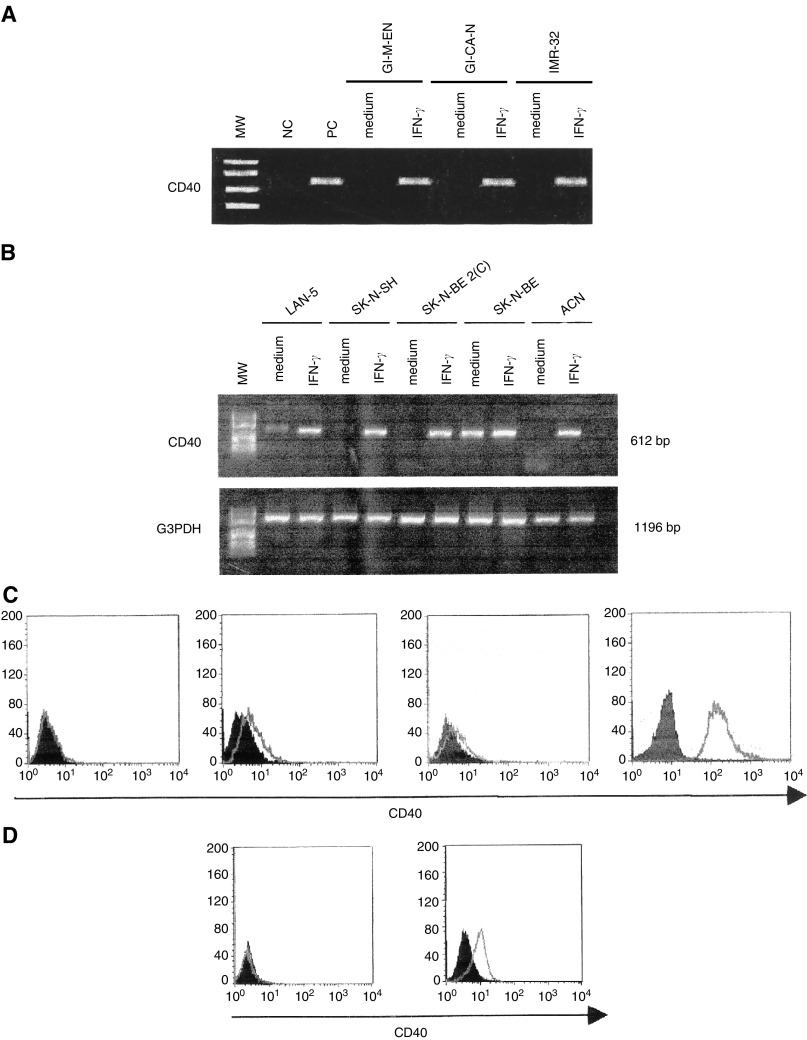
(**A**) Expression of CD40 mRNA, as assessed by RT–PCR, in GI-ME-N, GI-CA-N and IMR-32 cell lines cultured with medium alone or with human rIFN-*γ*. From left to right: MW=molecular weight markers; NC=negative control, represented by water in the place of cDNA; PC=positive control, represented by normal peripheral blood MNC stimulated with phytohaemagglutinin for 6 h; GI-ME-N cells cultured in the absence (medium) or presence of rIFN-*γ* (IFN-*γ*), GI-CA-N cells cultured in the absence (medium) or presence of rIFN-*γ* (IFN-*γ*), IMR-32 cells cultured in the absence (medium) or presence of rIFN-*γ* (IFN-*γ*). (**B**) Semiquantitative RT–PCR analysis for CD40 mRNA expression in LAN-5, SK-N-SH, SK-N-BE 2(C), SK-N-BE and ACN cells cultured in the absence (medium) or presence of rIFN-*γ* (IFN-*γ*) for 72 h. On the right side of each panel, the expected MW of the amplification products are shown. (**C**) Expression of CD40 in LAN-5 cells. From left to right: (1) cells cultured in medium for 72 h; (2) cells cultured in the presence of rIFN-*γ* for 72 h, and stained with a PE-conjugated CD40 mAb (open profile) or with a PE-conjugated isotype matched murine mAb of irrelevant specificity (dark profile); (3) cells cultured as in (2) and stained after permeabilisation with a PE-conjugated CD40 mAb (open profile) or with a PE-conjugated isotype matched murine mAb of irrelevant specificity (dark profile). The fourth histogram on the right side of the figure shows CD40 staining of Raji Burkitt's lymphoma cell line with a PE-conjugated CD40 mAb (open profile) or with a PE-conjugated isotype matched murine mAb of irrelevant specificity (dark profile). Cells were cultured 72 h in medium before staining. (**D**) Expression of surface CD40 in ACN cells transfected with empty plasmid (left) or with IFN-*γ* gene carrying plasmid (right). Cells were stained with a PE-conjugated CD40 mAb (open profiles) or with a PE-conjugated isotype matched murine mAb of irrelevant specificity (dark profiles).

GI-ME-N, GI-CA-N and IMR-32 cells showed *de novo* expression of the CD40 gene (Figure 2A

). Furthermore, the CD80 transcript was induced in LAN-5 cells, and B7H2 mRNA was detected in GI-ME-N cells (data not shown).

To investigate whether or not CD40 mRNA expression was upregulated by incubation with rIFN-*γ*, cells from the LAN-5, SK-N-SH, SK-N-BE 2(C), SK-N-BE and ACN cell lines were subjected to semiquantitative RT–PCR following 72 h culture with rIFN-*γ* or with medium alone (Figure 2B). A clear-cut increase of CD40 mRNA was observed after incubation of the LAN-5, SK-N-SH, SK-N-BE 2(C), and ACN cell lines, but not in the SK-N-BE cell line, with rIFN-*γ* (Figure 2B).

Next, CD40 surface expression was investigated by flow cytometry in rIFN-*γ* treated NB cell lines. ACN, GI-ME-N, LAN-5, SK-N-BE2(C), SK-N-SH and GI-CA-N cell lines were found to express surface CD40 on a minority of cells (range: 6–13%) (see Table 1

**Table 1:** Surface CD40 expression and apoptosis induction in neuroblastoma cell lines treated sequentially with rIFN-*γ* and rCD40L

Cell Lines	Percentage of CD40+ cells after IFN-*γ* treatment^a^	Percentage of CD40+ apoptotic cells after rCD40L treatment^b^
ACN	16	97.2
LAN-1	ND	ND
LAN-5	12	98.5
GI-LI-N	ND	ND
GI-ME-N	5	86.3
GI-CA-N	10	94.8
SK-N-SH	3	99.6
SK-N-BE	0	0
SK-N-BE 2(C)	14	89.7
IMR-32	0	0

a Cells were cultured for 72 h with 1000 U ml^−1^ rIFN-*γ* and stained with a CD40 mAb. Results are percentage of positive cells;

b Cells that had been pretreated with rIFN-*γ* as in (a) were washed and incubated for 48 h with rCD40L (1 *μ*g ml^−1^) and double stained with annexin and propidium iodide.

). One representative experiment carried out with the LAN-5 cell line is shown in Figure 2C, together with surface CD40 staining of Raji Burkitt's lymphoma cells shown as reference.

Surface CD40 was detected in cell lines that either displayed constitutive expression of the CD40 transcript (ACN, LAN-5, SK-N-BE2(C) and SK-N-SH), or expressed CD40 mRNA only following incubation with rIFN-*γ* (GI-ME-N and GI-CA-N).

Additional experiments were carried out to investigate whether a representative surface CD40− (i.e. GI-LI-N) and two representative CD40+ (i.e. SK-N-BE2(C) and LAN-5) cell lines contained intracellularly the CD40 protein following incubation with rIFN-*γ* or with medium alone. As shown in Figure 2C LAN-5 cells did not express intracellular CD40, as assessed by flow cytometry, unless they were incubated with rIFN-*γ*; similar results were obtained with the SK-N-BE2(C) cell line (not shown). In contrast, no intracellular CD40 protein was detected in GI-LI-N cells upon culture with either rIFN-*γ* or medium alone (not shown). These findings indicate that intracellular and surface CD40 expression are induced coordinately in rIFN-*γ*-responsive NB cell lines.

In view of the ability of rIFN-*γ* to upregulate CD40 expression on NB cells, ACN cells stably transfected with a plasmid containing the human IFN-*γ* gene or with the empty plasmid were tested for CD40 surface expression. The detailed characterisation of this cell line will be the matter of a separate report; suffices here to say that CD40 was consistently detected by flow cytometry on 50–60% of the cells transfected with the plasmid carrying the IFN-*γ* gene (Figure 2D, right panel), but not on cells transfected with the empty plasmid (Figure 2D, left panel). The proportion of CD40+ cells in IFN-*γ* gene transfected ACN cells remained stable over a 6 month period, with little fluctuations that did not exceed 10–15%.

### Expression of CD40, CD80, CD86, PD-1L, B7H2, OX40L and 4-1BBL in primary NB cells

First, costimulatory molecule gene expression was investigated in GD_2_+ neuroblasts isolated from four tumours (from one stage 1, one stage 2, one stage 3 and one stage 4 patients) by immunomagnetic bead manipulation. The GD_2_ disialoganglioside is a reliable surface marker of NB cells (Cheung *et al*, 1986; Mujoo *et al*, 1987).

**Figure 3:**
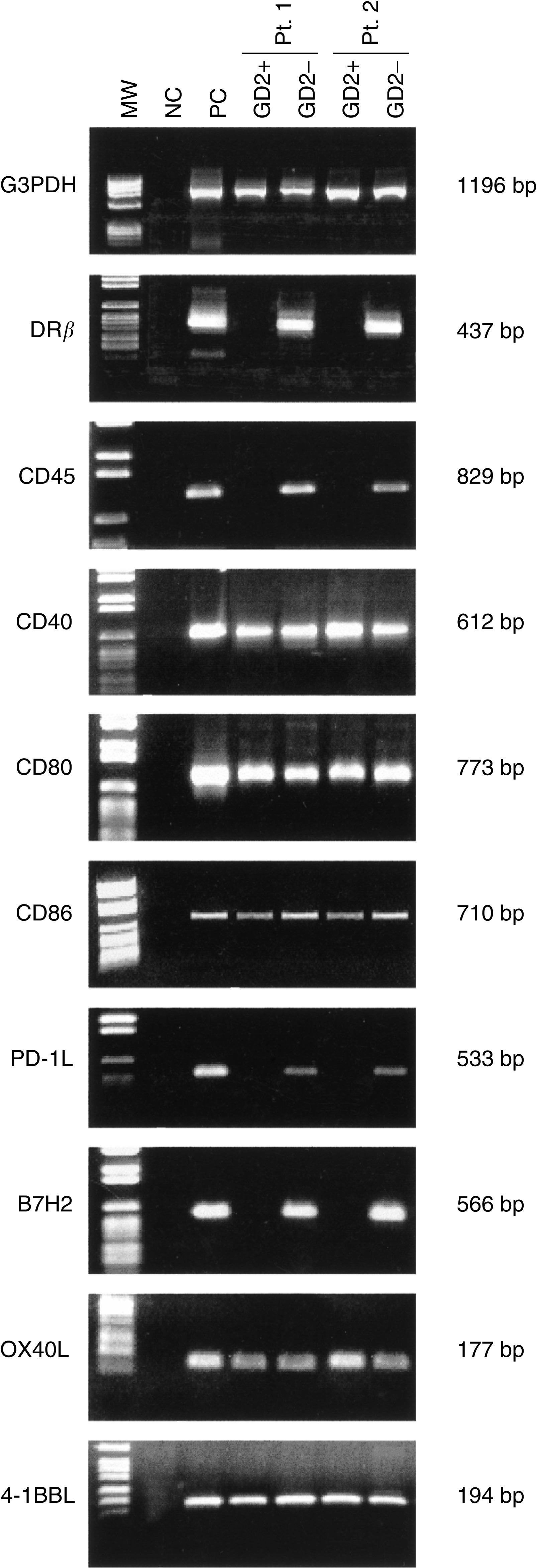
Costimulatory molecule gene expression in primary NB cells, as assessed by RT–PCR. The results of the experiments carried out with two tumours out of the four studied are shown. Neuroblasts were isolated as GD_2_+ cells by immunomagnetic bead manipulation. From left to right: MW=molecular weight markers; NC=negative control, represented by water in the place of cDNA; PC=positive control, represented by normal peripheral blood MNC stimulated with phytohaemagglutinin for 6 h; NB (GD_2_+) cells from patient 1 (Pt1); GD_2_− cell fraction from patient 1 (Pt 1); NB (GD_2_+) cells from patient 2 (Pt 2); GD_2_− cell fraction from patient 2 (Pt 2). The first upper panel shows the amplification product of the G3PDH housekeeping gene tested as control. On the right side of each panel, the expected MW of the amplified bands are shown.

The purity of the GD_2_+ cell suspensions was checked by RT–PCR with CD45 and HLA-DR*β* gene-specific primers (Figure 3

). Since these genes are not expressed in NB cells (Komada *et al*, 1998; Corrias *et al*, 2001), further experiments were carried out only with GD_2_+ cell suspensions that tested negative for CD45 and HLA-DR*β* mRNA expression. The latter transcripts were detected, as expected, in GD_2_− cell fractions.

Figure 3 shows the patterns of costimulatory molecule gene expression in GD_2_+ and GD_2_− cell fractions from two tumours (from a stage 2 and a stage 4 patient, respectively), representative of the four tested with similar results. Primary GD_2_+ neuroblasts were found to express CD40, CD80, CD86, OX40L and 4-1BBL, but not PD-1L or B7H2, mRNA. In contrast, all of the costimulatory gene transcripts were consistently detected in the GD_2_− cell fractions (Figure 3).

**Figure 4:**
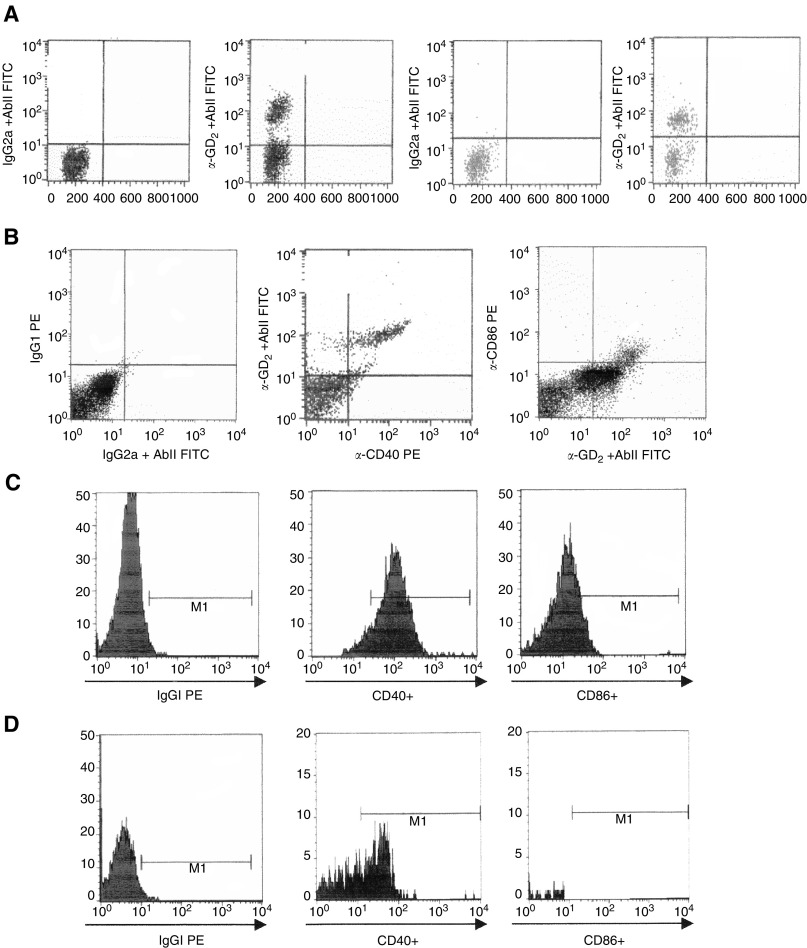
Surface expression of CD40 and CD86 on primary, GD_2_+ neuroblastoma cells, as assessed by flow cytometry. (**A**) Representative GD_2_ stainings of two BM samples shown as dot-plots. From left to right: cells stained with an irrelevant subclass-matched mAb (1,3) or with test mAb (2, 4) followed by incubation with FITC-conjugated secondary anti-mouse Ig antibodies. (**B**) Neuroblasts from one BM aspirate were double stained for GD_2_ and CD40 or CD86. Two-D dot-plots are shown. From left to right: cells were incubated with a PE-conjugated irrelevant subclass matched mAb (1) or PE-conjugated CD40 (2) or CD86 (3), followed by treatment with FITC-conjugated secondary anti-mouse Ig antibodies. (**C, D**) Neuroblasts from two BM aspirates were double stained for GD_2_ and CD40 or CD86. The profiles shown have been obtained by setting the gate on GD_2_+ neuroblasts. Left panels: no staining (<1.5% positive cells) was detected when an IgG2a antibody of irrelevant specificity (anti-GD_2_ control), followed by FITC-conjugated goat anti-mouse IgG2a antibodies, and a PE-conjugated IgG1 mAb (CD40 or CD86 control) of irrelevant specificity were tested together. Middle panels: neuroblasts were double stained with a GD_2_ mAb, followed by FITC-conjugated goat anti-mouse IgG2a antibodies, and with a PE-conjugated CD40 mAb. Right panels: Neuroblasts were double stained with a GD_2_ mAb, followed by FITC-conjugated goat anti-mouse IgG2a antibodies, and with a PE-conjugated CD86 mAb.

Flow cytometric analysis of six bone marrow (BM) aspirates (all from stage 4 patients) performed for diagnostic purposes was subsequently carried out by double staining for GD_2_ and CD40, CD80, CD86, OX-40L or 4-1BBL. All BM aspirates were found to contain GD_2_+ neuroblasts (Figure 4A

) with the following figures: BM1 50.8%, BM2 38.7%, BM3 65.7%, BM4 25.6%, BM5 30.2% and BM6 36.4%. GD_2_+ cells coexpressing CD40 were found in all BM samples and ranged from 10 to 65% (mean 35%). GD_2_+ cells coexpressing CD86 were detected in three BM samples (range 10–20%, mean 15%).

Figure 4B and C shows the results of immunophenotypic analyses of CD40 and CD86 expression on neuroblasts from three representative BM aspirates. As apparent, GD_2_+ neuroblasts expressed CD40 in both samples, whereas coexpression of CD86 was detected only in one case.

Neuroblasts tested always negative for CD80, OX40L or 41BBL staining.

### CD40L triggers apoptosis of NB cells expressing surface CD40

Previous studies have shown that ligation of CD40 on the surface of tumour cells by CD40L can induce inhibition of cell proliferation and apoptosis (Hirano *et al*, 1999; Ghamande *et al*, 2001; Bugajska *et al*, 2002; Szocinski *et al*, 2002). Therefore, subsequent experiments were addressed at investigating whether CD40+ NB cells were susceptible to CD40L-induced apoptosis.

First, IFN-*γ* pretreated ACN, GI-ME-N, LAN-5, SK-N-BE2(C), SK-N-BE, SK-N-SH, GI-CA-N and IMR-32 cell lines were incubated for 48 h with medium alone or with rCD40L, and tested for apoptosis by double staining for annexin V and propidium iodide (Koopman *et al*, 1994). As shown in Table 1, all the CD40+ cell lines were found to undergo apoptosis in the presence of rCD40L, while the two CD40− cell lines were unaffected by rCD40L treatment.

To better investigate the kinetics of CD40L-induced apoptosis and the mechanism involved, subsequent experiments were carried with IFN-*γ* gene-transfected ACN cells. To this end, IFN-*γ* gene-transfected ACN cells were incubated for 5–72 h with medium alone, or rCD40L, or NIH-3T3 murine fibroblasts, either transfected with the human CD40L gene, or with the empty vector.

**Figure 5:**
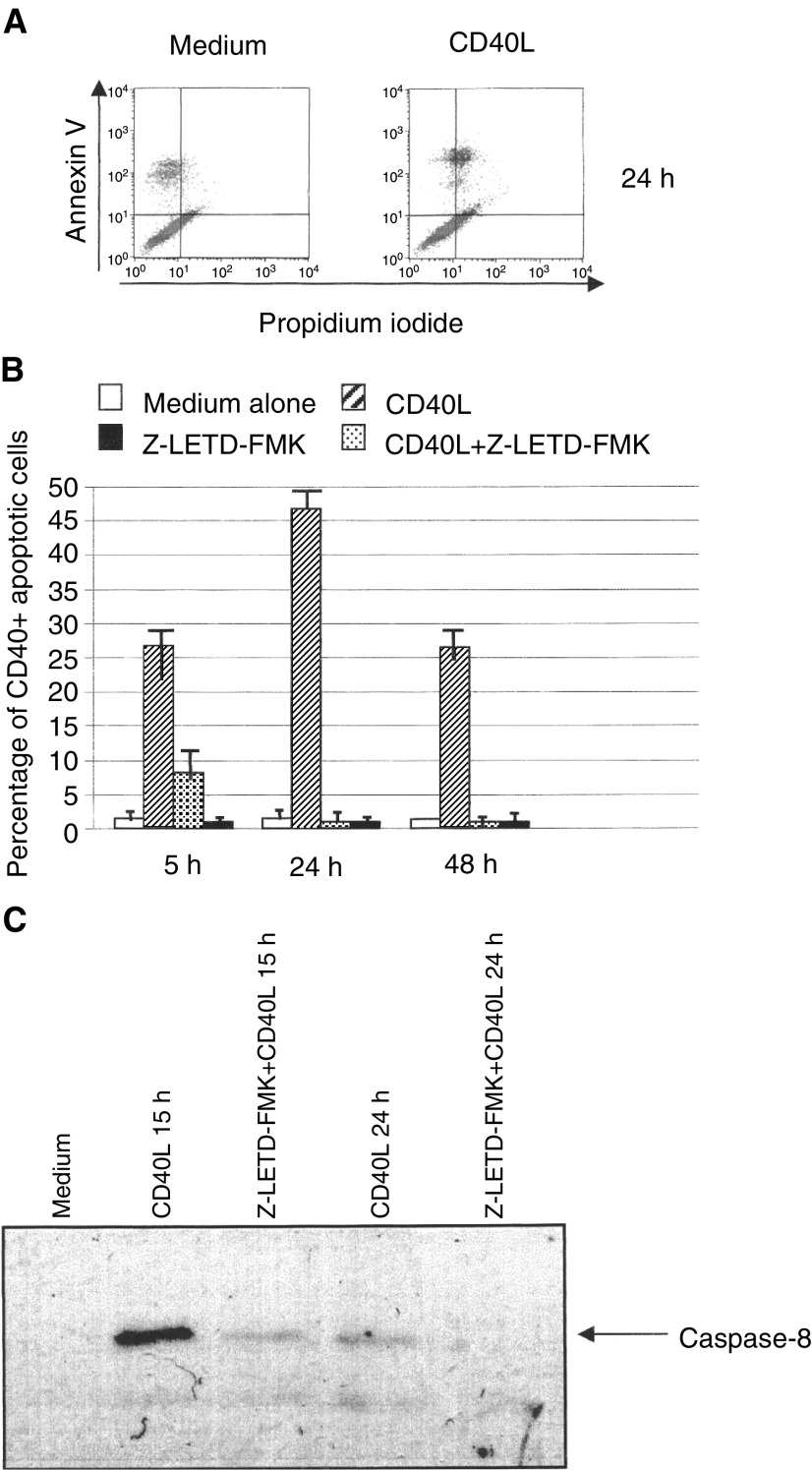
Apoptosis of IFN-*γ* gene-transfected ACN cells following incubation with rCD40L and their rescue upon exposure to the selective caspase-8 inhibitor Z-LETD-FMK. (**A**) Quadrant analysis of annexin V *vs* propidium iodide staining of IFN-*γ* gene transfected ACN cells incubated 24 h with rCD40L. One representative experiment is shown. (**B**) IFN-*γ* gene-transfected ACN cells were cultured for 5, 24 and 48 h with medium alone, rCD40L, rCD40L together with Z-LETD-FMK, or Z-LETD-FMK alone. The results, which represent the means from four independent experiments, are shown as percent CD40+ cells undergoing apoptosis ±s.d. These values were calculated as follows. The percentages of apoptotic cells in CD40L containing cultures, detected by double staining with annexin V and propidium iodide, were divided by the percentage of CD40+ cells determined at any time interval in control cultures and multiplied by 100. (**C**) Western blot analysis performed on IFN-*γ* gene transfected ACN cells incubated with medium or rCD40L for 15 or 24 h, in the absence or presence of the caspase-8 inhibitor Z-LETD-FMK. The band corresponding to activated 17 kDa caspase-8 is indicated by the arrow on the right side of the figure.

A dot-plot representative of these experiments is shown in Figure 5B

, where cell apoptosis was tested after 24 h culture with rCD40L.

Figure 5B shows the results of time course experiments in which IFN-*γ* gene-transfected ACN cells were cultured in the presence or absence of rCD40L for 5, 24 or 48 h. Results are expressed as mean percentages (from four independent experiments) of CD40+ cells undergoing apoptosis. These values were obtained by dividing the percentage of apoptotic cells in CD40L containing cultures by the percentage of CD40+ cells in control cultures for each time point. As apparent, apoptosis was detected in IFN-*γ* gene-transfected ACN cells that had been cultured with rCD40L for 5, 24 and 48 h. At 72 h, apoptotic cells were no longer found in these cultures (not shown). Cells kept in medium alone for the same time intervals showed only minimal levels of apoptosis (Figure 5B).

The above experiments were repeated by incubating for the same times IFN-*γ* gene-transfected ACN cells with NIH-3T3 murine fibroblasts, either transfected with the human CD40L gene or with the empty vector for the same times. Apoptosis of ACN cells was detected with the same kinetics as above following incubation with the former, but not the latter, transfectant (data not shown). These results indicate that soluble and insoluble CD40L were equally effective at inducing apoptosis of IFN-*γ* gene-transfected ACN cells.

### Apoptosis of CD40+ NB cells induced by CD40L is caspase-8 dependent

CD40 belongs to the TNF receptor superfamily, whose components trigger cell apoptosis through caspase-8 activation (Ashkenazi and Dixit, 1998; Grell *et al*, 1999; Eliopoulos *et al*, 2000). We therefore investigated whether this mechanism operated also in CD40-expressing NB cells. IFN-*γ* gene-transfected ACN cells were treated overnight with the recently developed caspase-8 selective inhibitor Z-LE(Ome)-TD(Ome)-FMK (Z-LETD-FMK) (Ahmad and Shi, 2000), before being incubated with rCD40L. Z-LETD-FMK was then added every 24 h to the cultures and apoptosis was assessed by flow cytometry.

As shown in Figure 5B, incubation of IFN-*γ* gene-transfected ACN cells with Z-LETD-FMK decreased apoptosis induced by CD40L after 5 h by approximately 70% and abrogated programmed cell death in cells tested at 24 and 48 h. Z-LETD-FMK alone did not cause apoptosis of IFN-*γ* gene-transfected ACN cells cultured up to 48 h (Figure 5B). The results are shown as mean percentages (from four independent experiments) of CD40+ cells undergoing apoptosis.

Finally, involvement of caspase-8 in CD40L-induced apoptosis of NB cells was demonstrated by Western blot experiments in which IFN-*γ* gene-transfected ACN cells were cultured with rCD40L or medium in the presence or absence of Z-LETD-FMK for 15 or 24 h (Figure 5C). After 15 h culture with rCD40L, the specific 17 kDa protein corresponding to active caspase-8 was clearly detectable in IFN-*γ* gene-transfected ACN cells. Treatment with Z-LETD-FMK strongly reduced the expression of activated caspase-8. After 24 h treatment with rCD40L, the band corresponding to activated caspase-8 was faint; addition of Z-LETD-FMK to the cultures led to its complete disappearance (Figure 5C). Taken together these findings demonstrate the direct role of caspase-8 in rCD40L-induced apoptosis of CD40+ NB cell lines.

## DISCUSSION

Expression of seven costimulatory molecules (CD40, CD80, CD86, PD-1L, B7H2, OX40L and 4-1BBL) has been investigated in a panel of human NB cell lines and in primary NB cells. The rationale for this study came from the following: (i) costimulatory molecules are poorly expressed on the surface of malignant cells (Hurwitz *et al*, 2000), (ii) transfer of some costimulatory molecule coding genes in tumour cells leads to tumour rejection *in vivo* (Katsanis *et al*, 1995; Enomoto *et al*, 1997) and (iii) virtually no information is available as to costimulatory molecule expression in human NB (Uçar *et al*, 1995).

Costimulatory gene transcripts were expressed constitutively in the majority of cell lines, but the CD40, CD80, CD86, OX40L and 4-1BBL proteins were not detected on the cell surface. Primary NB cells displayed a similar profile of gene expression, but PD-1L and B7H2 transcripts were not detected.

CD86 was detected on the surface of neuroblasts from three out of six BM cell suspensions tested. CD80 and/or CD86 expressed on neoplastic cells may induce NK cell activation, possibly because of the triggering of CD80/CD86 ligands alternative to CD28 or CTLA-4 (Wilson *et al*, 1999). In addition, targeting of CD86 and/or CD80 in experimental tumours, even of low immunogenicity, was found to promote antitumour CTL responses (Moro *et al*, 1999). Thus, our preliminary observation on CD86 expression in some NB cases deserves further investigation.

The most important finding of this study is the constitutive expression of CD40 mRNA and protein in all of the tumour samples tested. In contrast, the majority of NB cell lines were found to express constitutively CD40 mRNA, but surface CD40 was detected on a minority of cells in the individual cell lines, only following culture with rIFN-*γ*. Additional experiments were performed to investigate whether lack of expression of surface CD40 in some NB cell lines following treatment with rIFN-*γ* was related to defects in CD40 protein transport. The finding that surface CD40 expression was paralleled by accumulation of intracellular CD40 protein in rIFN-*γ*-responsive cell lines, whereas no intracellular CD40 was detected in unresponsive cell lines, suggest that the inability of the latter cell lines to express the CD40 protein upon incubation with rIFN-*γ* was not because of defects in protein trafficking.

To get further insight into the mechanisms involved in the rIFN-*γ*-mediated induction of CD40 protein in NB cell lines, CD40 mRNA expression was evaluated by semiquantitative RT–PCR in cell cultured with rIFN-*γ* or medium alone. These experiments showed that surface CD40+ cell lines displayed an evident upregulation of CD40 mRNA following incubation with rIFN-*γ*, whereas a surface CD40− cell line did not, suggesting that regulation of CD40 expression occurred mainly at the transcriptional level. However, additional, post-transcriptional mechanisms may be involved, since IMR-32 cells, that acquired *de novo* expression of CD40 mRNA following incubation with rIFN-*γ*, failed to express surface CD40.

The discrepancies in CD40 expression between NB cell lines and fresh tumour cells may be related to the presence of *in vivo* factors (e.g. IFN-*γ*) capable of upregulating surface CD40 in the tumour microenvironment.

CD40 expression is a common feature of haematopoietic and nonhaematopoietic tumours (Hirano *et al*, 1999; Ghamande *et al*, 2001; Bugajska *et al*, 2002; Szocinski *et al*, 2002). Previous studies have shown that interaction of tumour cell-associated CD40 with CD40L can lead to different consequences: (i) CD40 triggering induces upregulation of HLA and adhesion molecules on the surface of tumour cells (Ahmad and Shi, 2000), thus enhancing their immunogenicity, and (ii) exposure of neoplastic cells to soluble CD40L results into inhibition of cell proliferation and induction of cell death by apoptosis (Hirano *et al*, 1999; Ghamande *et al*, 2001; Bugajska *et al*, 2002; Szocinski *et al*, 2002). Furthermore, soluble CD40L was found to induce tumour regression in SCID mice implanted with human breast cancer cells (Hirano *et al*, 1999).

The functional role of surface CD40 in neuroblastoma cells was investigated using both IFN-*γ*-treated cell lines and the ACN cell line transfected with a plasmid carrying the human IFN-*γ* gene. In these experiments, cells were incubated with soluble or insoluble CD40L and tested for apoptosis. All CD40+ neuroblastoma cells underwent massive apoptosis following 48 h culture with CD40L.

Since CD40 belongs to the TNF receptor superfamily that triggers apoptosis by caspase-8 activation (Ashkenazi and Dixit, 1998; Grell *et al*, 1999; Eliopoulos *et al*, 2000), experiments were performed in which CD40L-induced apoptosis of ACN cells was investigated in the presence or absence of Z-LETD-FMZ, a selective inhibitor of caspase-8 (Ahmad and Shi, 2000). It was found that apoptosis of CD40+ ACN cells cultured with CD40L was abrogated by Z-LETD-FMZ, thus pointing to the involvement of caspase-8 in our experimental model. The latter conclusion was directly supported by Western blot experiments showing activation of caspase-8 in NB cells following CD40–CD40L interaction and its block by exposure to the Z-LE-TD-FMK inhibitor.

Previously, it was shown that the CD40 cytoplasmic C terminus lacks a death domain homology with the cytotoxic members of the TNF-R superfamily, such as Fas, TNF-R1 and TNF-related apoptosis-inducing ligand (TRAIL) (Ashkenazi and Dixit, 1998; Grell *et al*, 1999; Eliopoulos *et al*, 2000). CD40L-induced apoptosis in neoplastic cells was found to depend on the membrane proximal domain of the molecule (Eliopoulos *et al*, 2000) and to involve endogenous production of TNF in the target cell, leading to autotropic or paratropic activation of TNF-R1 and, eventually, to TNF-R1-mediated cytotoxicity (Grell *et al*, 1999). In this connection, human NB cell lines were reported to express surface TNF-R1 and TNF-R2, as well as TNF mRNA (Goillot *et al*, 1992; Montaldo *et al*, 1994). Thus, it is conceivable that NB cell apoptosis induced by CD40L takes place through the TNF/TNF-R1 pathway identified in other experimental models.

Recently, caspase-8 gene expression was found to be reduced or absent in neoplastic cells from patients with metastatic NB (Hopskins-Donaldson *et al*, 2000; Teitz *et al*, 2001). This feature has been related to methylation of caspase-8 gene promoter regions (Teitz *et al*, 2000; Banelli *et al*, 2002) or, more rarely, to deletion of the caspase-8 gene (Teitz *et al*, 2000). In the former case, demethylating agents, such as 5-aza-2′-deoxycytidine, or IFN-*γ* can restore caspase-8 gene expression in NB cells (Fulda *et al*, 2001; Banelli *et al*, 2002; Fulda and Debatin, 2002), whereas this cannot happen in the latter case. Thus, the potential therapeutic use of rCD40L in NB patients may depend on preliminary *in vitro* assessment of caspase-8 activation in tumour cells under baseline conditions and following culture with demethylating agents or IFN-*γ*. Thereafter, combination therapy with either of the latter molecules and rCD40L may be envisaged.

Triggering of dendritic cell-associated CD40 leads to increased production of IL-12, which activates NK cells and drives T helper 1 lymphocyte responses (Trinchieri, 1998). Notably, this network of cell interactions has been shown to be involved in the regression of murine metastatic neuroblastoma (Turner *et al*, 2001). Thus, CD40 may represent a novel therapeutic, multifunctional target in NB because of its expression on both tumour cells and professional antigen-presenting cells. Since, in the present study, surface CD40 was detected on a fraction of neuroblasts from the individual tumours, CD40L-induced apoptosis of malignant cells and immune activation may cooperate in the elimination of CD40+ and CD40− neuroblasts, as already shown in a different neoplastic model (Dotti *et al*, 2002).

A trimeric recombinant form of soluble CD40L, recently tested in Phase I studies with lymphoma or solid tumour bearing patients, showed limited toxicity, indicating the potential feasibility of this therapeutic approach in NB patients (Vonderheide *et al*, 2001a, 2001b).

## MATERIALS AND METHODS

### Patient samples

This investigation was performed after approval by a local institutional review board. Four NB tumours were obtained from the local pathologist (CG). Disease stage according to the International Neuroblastoma Staging System (Brodeur *et al*, 1998) was: stage 1 (one case), stage 2 (one case), stage 3 (one case) and stage 4 (one case). Aliquots of BM aspirates performed for diagnostic purposes were from six patients with stage 4 disease. These samples contained at least 25% infiltrating neuroblasts, as assessed by morphological criteria and anti-GD_2_ staining.

### Cell separation and culture

Solid tumours were minced in RPMI 1640 medium (Seromed-BiochromKG, Berlin, Germany) supplemented with 10% fetal calf serum (Seromed) (RPMI-FCS) and subjected to gravity sedimentation for 2 min to remove large debris. Supernatants were used for further experiments. Bone marrow aspirates were depleted of erythrocytes by osmotic lysis, washed twice in RPMI–FCS and tested.

Neuroblastoma cells were isolated as follows. Cells were first incubated with an anti-GD_2_ mAb and then positively selected by immunomagnetic beads coated with anti-mouse immunoglobulin antibodies, according to the instructions of the manufacturer (Immunotech, Marseille, France). The source of the anti-GD_2_ mAb (IgG2a) was the supernatant of the ME361-S2a murine hybridoma, purchased from ATCC, Manassas, VA, USA.

The ACN, SK-N-BE, SK-N-BE2(C), SK-N-SH, LAN-1, LAN-5, GI-LI-N, GI-ME-N, GI-CA-N and IMR-32 NB cell lines, whose features are described in Thiele (1999), or IFN-*γ* gene-transfected ACN cells were cultured in RPMI–FCS for the indicated times. IFN-*γ* gene-transfected ACN cells were generated by one of us (MVC) in collaboration with Dr Silvano Ferrini, IST, Genova. In some experiments, NB cell lines were incubated with or without rIFN-*γ* (1000 IU ml^−1^) (Imuchin®, Boehringher Ingelheim Italia, Florence, Italy) for 72 h before undergoing molecular or immunophenotypic studies. The rIFN-*γ* concentration and the culture time were selected on the ground of previous studies (Montaldo *et al*, 1997).

### Monoclonal antibodies

Flow cytometric analysis was carried out as reported (Airoldi *et al*, 2000). Neuroblastoma cells were always stained in parallel with positive controls, that is, the Colo 205 cell line for OX40L and 4-1BBL staining (Salih *et al*, 2000), and the Raji cell line for CD40, CD80 and CD86 staining.

The following murine mAbs were used for single or double staining: PE-conjugated CD40, FITC-conjugated CD80 and PE-conjugated CD86 mAbs, all from Diaclone (Diaclone SA, Besançon, France) and anti-GD_2_ from ATCC (see above). Cells stained with the latter mAb were washed and incubated with a PE- or FITC-conjugated goat anti-mouse IgG subclass antiserum (Dako, Glostrup, Denmark), depending on the partner mAb used in double staining experiments. Controls for CD40, CD80 and CD86 were fluorochrome-conjugated, isotype-matched murine mAbs of irrelevant specificity. Control for anti-GD_2_ staining was purified murine IgG2a (Southern Biotechnology Associates, Birmingham, AL, USA) of irrelevant specificity.

In addition, the rhOX40:Fc and rh4-1BB:Fc (Alexis Corp., Del Mar, CA, USA) fusion proteins, in which the OX40 and 4-1BB proteins are fused with the Fc portion of human IgG1, were used for single or double staining. Cells were incubated with the fusion proteins or with the purified Fc portion of human IgG1 (Alexis Biochemicals, Lausanne, Switzerland) as control, washed and stained with FITC-conjugated goat anti human IgG (Fc portion-specific) antibodies (Alexis).

In some experiments, GI-LI-N, SK-N-BE2(C) and LAN-5 cell lines that had been preincubated 72 h with rIFN-*γ* were washed and stained with the CD40 mAb for 30 min at 4°C in the dark. Cells were then washed in PBS containing 1% FCS and fixed in 4% para-formaldehyde for 20 min at 4°C in the dark. Afterwards, the cells were washed twice with permeabilisation buffer (PBS, 1% FCS, 0.1% saponin, Sigma Aldrich, Milano, Italy) and stained with FITC-conjugated goat anti-mouse antiserum specific for the appropriate murine IgG subclass or with a FITC-conjugated, goat antiserum of irrelevant specificity for 30 min at 4°C in the dark. Cells were washed in staining buffer and analysed by flow cytometry (FACScan-BD Biosciences-Mountain View, CA, USA).

### Reverse transcription–polymerase chain reaction and sequencing

RNA was extracted from freshly isolated or cultured cells using RNeasy Mini Kit from Qiagen (Qiagen GmbH, Hilden, Germany) and subjected to RT–PCR as reported (Turner *et al*, 2001). Primer sequences and profiles of amplification were the following: G3PDH 5′ ACATCGCTCAGAACACCTATGG and 3′ GGGTCTACATGGCAACTGTGAG, CD45 5′ CCTACAGACCCAGTTTCC and 3′ GGCAATCTTTTTCTGTCT, HLA-DR*β* 5′ CTCCAGCATGGTGTGTCTGA and 3′ GGAGGTTGTGGTGCTGCAGG, CD40 5′ CTGGGGCTGCTTGCTGAC and 3′ TCCTGGGGTTCCTGCTTG, CD80 5′ GGTCTTTCTCACTTCTGTTC and 3′ CTTTCCCTTCTCAATCTCTC, CD86 5′ ACACGGAGGCAGGGAACA and 3′ GGAAAATGCTCTTGCTTGGT, PD-1L 5′ GGGAAATGGAGGATAAGA and 3′ AGGATGTGCCAGAGGTAG, B7H2 5′ CCGAGCCCTGATGTCACC and 3′ CCGCCACGACCACAAGCA, 4-1BBL 5′ ACAAAGAGGACACGAAGGAG and 3′ GGAGGAGGCGGGTGGCAGGT, OX40L 5′ TCAACATTAGCCTTCATTACC and 3′ GAATCAGTTCTCCGCCATTCA. Amplification profile was 94°C for 1 min, annealing 60°C (G3PDH), 49°C (CD45), 55°C (HLA-DR*β*), 57°C (CD40), 52°C (CD80), 54°C (CD86), 51°C (PD-1L and OX40L), 58°C (B7H2), 59°C (4-1BBL) for 1 min and extension at 72°C for 1 min. Each cycle of amplification was repeated 35 times.

A volume of 10 *μ*l of each sample were electrophoresed through a 1% agarose gel containing ethidium bromide. The specificity of amplification products was checked by confirming the known base-pair sequence length and by sequencing.

Direct sequencing of PCR products was performed with the use of the Dye Terminator Cycle Sequencing Kit (ABI PRISM; Perkin-Elmer Applied Biosystem, Norwalk, CT, USA). Sequences were resolved and analysed on the ABI 373A Sequence Apparatus (Perkin-Elmer Applied Biosystem).

For semiquantitative RT–PCR analysis, nonsaturating conditions were used, that is, 1/5 of cDNA previously used and amplifications with CD40 or G3PDH-specific primers were performed for 30 cycles.

### Assay for assessment of apoptosis

ACN cells transfected with the plasmid carrying the IFN-*γ* gene or with the empty plasmid were cultured in six-well plates (Costar, Cambridge, MA, USA) for 5–72 h: (i) in the absence or presence of rCD40L (Immunex, Seattle, WA, USA) at 1 *μ*g ml^−1^. This concentration was selected on the ground of preliminary titration experiments in which rCD40L was tested in the range of 0.1–100 *μ*g ml^−1^, or (ii) NIH3T3 murine fibroblasts transfected with the pIRES-neo bicystronic plasmid vector carrying the human CD40L gene or with the empty vector. These cultures were set up at a 4 : 1 NB/CD40 L or control transfectant cell ratio. The latter transfectants were kindly provided by Dr Franco Fais, Department of Experimental Medicine, University of Genova, Italy.

Cells were harvested by brief trypsinisation and the proportion of apoptotic cells was assessed by flow cytometry using an annexin V–FITC apoptosis kit according to the manufacturer's instruction (Bender MedSystems, Vienna, Austria). In this assay, cells double staining for annexin and propidium iodide are apoptotic, whereas cells single positive for propidium iodide staining are necrotic. In the experiments in which murine fibroblasts were cocultured with neuroblasts, the latter cells were first gated on the basis of their larger size and then analysed by double staining for annexin and propidium iodide. Gated cells were uniformly GD_2_+, as assessed by flow cytometry.

For inhibition of apoptosis studies, IFN-*γ* gene-transfected ACN cells were preincubated overnight with the selective caspase-8 inhibitor, Z-LE(Ome)-TD(Ome)-FMK (Alexis) at the final concentration of 20 *μ*M before adding rCD40L. The cultures were supplemented with Z-LETD-FMK every 24 h. Cells were harvested and assessed for apoptosis by flow cytometry as described above.

### Total extracts and Western blot analysis

Cells were incubated for 30 min on ice with lysis buffer containing 20 mM HEPES, 150 mM NaCl, 10% glycerol, 0.5%. NP-40, 1 mM EDTA, 2.5 mM DTT, 10 *μ*g ml^−1^ aprotinin, 10 *μ*g ml^−1^ leupeptin, 1 *μ*g ml^−1^ pepstatin A, 1 mM PMSF and 1 mM Na_3_VO_4_, all from Sigma-Aldrich. During this time interval, cells were subjected to vortex mixing every 5 min. Thereafter, lysates were centrifuged at 12 000 r.p.m. for 5 min at 4°C and supernatants quantitated by the BCA kit assay (Pierce, Rockford, IL, USA). Equal amounts of protein (10 *μ*g) were loaded on 8% SDS–polyacrylamide gel and boiled 3 min before application. Gel was blotted onto Protean nitrocellulose membrane (Schleicher & Schuell, Dassel, Germany) for 1 h at 100 V. Blots were incubated in a blocking buffer containing BSA and 0.5% Tween-20 in Tris-buffered saline (TBS) for 1 h, followed by incubation with goat anti-human Caspase-8 antiserum (Santa Cruz Biotechnology Inc, Santa Cruz, CA, USA). After three washings in TBS–Tween, blots were incubated for 1 h with rabbit anti-goat Ig conjugated with horseradish peroxidase (Santa Cruz) at the final concentration of 50 ng ml^−1^ in TBS–Tween containing 1% BSA. Detection was performed by enhanced chemiluminescence (ECL, Pierce, NE, USA).

## References

[bib1] Ahmad M, Shi Y (2000) TRAIL-induced apoptosis of thyroid cancer cells: potential for therapeutic intervention. Oncogene 19: 3363–3371 10918593 10.1038/sj.onc.1203679

[bib2] Aicher A, Hayden-Ledbetter M, Brady WA, Pezzutto A, Richter G, Magaletti D, Buckwalter S, Ledbetter JA, Clark EA (2000) Characterization of human inducible costimulatory ligand expression and function. J Immunol 164: 4689–4696 10779774 10.4049/jimmunol.164.9.4689

[bib3] Airoldi I, Gri G, Marshall JD, Corcione A, Facchetti P, Guglielmino R, Trinchieri G, Pistoia V (2000) Expression and function of IL-12 and IL-18 receptors on human tonsillar B cells. J Immunol 165: 6880–6888 11120812 10.4049/jimmunol.165.12.6880

[bib4] Akiba H, Oshima H, Takeda K, Atsuta M, Nakano H, Nakajima A, Nohara C, Yagita H, Okumura K (1999) CD28-independent costimulation of T cells by OX40 ligand and CD70 on activated B cells. J Immunol 162: 7058–7066 10358148

[bib5] Ashkenazi A, Dixit VM (1998) Death receptors: signaling and modulation. Science 281: 1305–1308 9721089 10.1126/science.281.5381.1305

[bib6] Banelli B, Casciano I, Croce M, Vinci AD, Gelvi I, Pagnan G, Brignole C, Allemanni G, Ferrini S, Ponzoni M, Romani M (2002) Expression and methylation of CASP8 in neuroblastoma: identification of a promoter region. Nat Med 8: 1333–1335 12457155 10.1038/nm1202-1333

[bib7] Brodeur GM, Seeger RC, Barret A, D'Angio G, De Bernardi B, Evans AE, Haase J (1988) International criteria for diagnosis, staging, and response to treatment in patients with neuroblastoma. J Clin Oncol 6: 1874–1881 3199170 10.1200/JCO.1988.6.12.1874

[bib8] Bugajska U, Georgopoulos NT, Southgate J, Johnson PW, Graber P, Gordon J, Selby PJ, Trejdosiewicz LK (2002) The effect of malignant transformation on susceptibility of human urothelial cells to CD40-mediated apoptosis. J Natl Cancer Inst 18: 1381–1395 10.1093/jnci/94.18.1381 12237284

[bib9] Cannons JL, Lau P, Ghumman B, DeBenedette MA, Yagita H, Okumura K, Watts TH (2001) 4-1BB ligand induces cell division, sustains survival, and enhances effector function of CD4 and CD8 T cells with similar efficacy. J Immunol 167: 1313–1324 11466348 10.4049/jimmunol.167.3.1313

[bib10] Chambers CA, Kuhns MS, Egen JG, Allison JP (2001) CTLA-4-mediated inhibition in regulation of T cell responses: mechanisms and manipulation in tumor immunotherapy. Annu Rev Immunol 19: 565–594 11244047 10.1146/annurev.immunol.19.1.565

[bib11] Chambers CA (2001) The expanding world of co-stimulation: the two-signal model revisited. Trends Immunol 22: 217–223 11274928 10.1016/s1471-4906(01)01868-3

[bib12] Cheung NK, Von Hoff DD, Strandjord SE, Coccia PF (1986) Detection of neuroblastoma cells in bone marrow using GD2 specific monoclonal antibodies. J Clin Oncol 4: 363–369 3081691 10.1200/JCO.1986.4.3.363

[bib13] Corrias MV, Occhino M, Croce M, De Ambrosis A, Pistillo MP, Bocca P, Pistoia V, Ferrini S (2001) Lack of HLA-class I antigens in human neuroblastoma cells: analysis of its relationship to TAP and tapasin expression. Tissue Antigens 57: 110–117 11260505 10.1034/j.1399-0039.2001.057002110.x

[bib14] Dong C, Juedes AE, Temann UA, Shresta S, Allison JP, Ruddle NH (2001) ICOS co-stimulatory receptor is essential for T-cell activation and function. Nature 409: 97–101 11343121 10.1038/35051100

[bib15] Dotti G, Savoldo B, Yotnda P, Rill D, Brenner MK (2002) Transgenic expression of CD40 ligand produces an *in vivo* antitumor immune response against both CD40(+) and CD40(−) plasmacytoma cells. Blood 1: 200–207 10.1182/blood.v100.1.200 12070028

[bib16] Eliopoulos AG, Davies C, Knox PG, Gallagher NJ, Afford SC, Adams DH, Young LS (2000) CD40 induces apoptosis in carcinoma cells through activation of cytotoxic ligands of the tumor necrosis factor superfamily. Mol Cell Biol 20: 5503–5515 10891490 10.1128/mcb.20.15.5503-5515.2000 PMC86001

[bib17] Enomoto A, Kato K, Yagita H, Hokumura K (1997) Adoptive transfer of cytotoxic T lymphocytes induced by CD86-transfected tumor cells suppresses multi-organ metastases of C1300 neuroblastoma in mice. Cancer Immunol Immunother 44: 204–210 9222278 10.1007/s002620050374 PMC11037674

[bib18] Freeman GJ, Long AJ, Iwai Y, Bourque K, Chernova T, Nishimura H, Fitz JL, Malenkovich N, Okazaki T, Byrne MC, Horton HF, Fouser L, Carter L, Ling V, Bowman MR, Carreno BM, Collins M, Wood CR, Honjo T (2000) Engagement of the PD-1 immunoinhibitory receptor by a novel B7 family member leads to negative regulation of lymphocyte activation. J Exp Med 192: 1027–1034 11015443 10.1084/jem.192.7.1027 PMC2193311

[bib19] Fulda S, Debatin KM (2002) IFN gamma sensitizes for apoptosis by upregulating caspase-8 expression through the Stat1 pathway. Oncogene 21: 2295–2308 11948413 10.1038/sj.onc.1205255

[bib20] Fulda S, Kufer MU, Meyer E, van Valen F, Dockhorn-Dworniczak B, Debatin KM (2001) Sensitization for death receptor- or drug-induced apoptosis by re-expression of caspase-8 through demethylation or gene transfer. Oncogene 20: 5865–5877 11593392 10.1038/sj.onc.1204750

[bib21] Ghamande S, Hylander BL, Oflazoglu E, Lele S, Fanslow W, Repasky EA (2001) Recombinant CD40 ligand therapy has significant antitumor effects on CD40-positive ovarian tumor xenografts grown in SCID mice and demonstrates an augmented effect with cisplatin. Cancer Res 20: 7556–7562 11606394

[bib22] Goillot E, Combaret V, Ladenstein R, Baubet D, Blay JY, Philip T, Favrot MC (1992) Tumor necrosis factor as an autocrine growth factor for neuroblastoma. Cancer Res 11: 3194–3200 1317260

[bib23] Grell M, Zimmermann G, Gottfried E, Chen CM, Grunwald U, Huang DCS, Lee YHW, Durkop H, Engelmann H, Scheurich P, Wajant H, Strasser A (1999) Induction of cell death by tumor necrosis factor (TNF) receptor 2, CD40 and CD30: a role for TNF-R1 activation by endogenous membrane-anchored TNF. EMBO J 18: 3034–3043 10357816 10.1093/emboj/18.11.3034 PMC1171385

[bib24] Hirano A, Longo DL, Taub DD, Ferris DK, Young LS, Eliopoulos AG, Agathanggelou A, Cullen N, Macartney J, Fanslow WC, Murphy WJ (1999) Inhibition of human breast carcinoma growth by a soluble recombinant human CD40 ligand. Blood 93: 2999–3007 10216096

[bib25] Hopskins-Donaldson S, Bodmer JL, Bourloud KB, Brognara CB, Tschopp J, Gross N (2000) Loss of caspase-8 expression in highly malignant human neuroblastoma cells correlates with resistance to tumor necrosis factor-related apoptosis-inducing ligand-induced apoptosis. Cancer Res 60: 4315–4319 10969767

[bib26] Hurwitz AA, Kwon ED, van Elsas A (2000) Costimulatory wars: the tumor menace. Curr Opin Immunol 12: 589–596 11007364 10.1016/s0952-7915(00)00147-3

[bib27] Katsanis E, Xu Z, Bausero MA, Dancisak BB, Gorden KB, Davis G, Gray GS, Orchard PJ, Blazar BR (1995) B7-1 expression decreases tumorigenicity and induces partial systemic immunity to murine neuroblastoma deficient in major histocompatibility complex and costimulatory molecules. Cancer Gene Ther 2: 39–46 7542553

[bib28] Komada Y, Zhang XL, Zhou YW, Inaba H, Deguchi T, Azuma E, Sakurai M (1998) Flow cytometric analysis of peripheral blood and bone marrow for tumor cells in patients with neuroblastoma. Cancer 82: 591–599 9452279

[bib29] Koopman G, Reutelinsperger CP, Kuijten GAM, Keehnen RMJ, Pals ST, van Oers MHJ (1994) Annexin V for flow cytometric detection of phosphatidylserine expression on B cell undergoing apoptosis. Blood 84: 1415–1420 8068938

[bib30] Lenschow DJ, Walunas TL, Bluestone JA (1996) CD28/B7 system of T-cell costimulation. Annu Rev Immunol 14: 233–258 8717514 10.1146/annurev.immunol.14.1.233

[bib31] Liu X, Bai XF, Wen J, Gao JX, Liu J, Lu P, Wang Y, Zheng P, Liu Y (2001) B7H costimulates clonal expansion of, and cognate destruction of tumor cells by CD8^+^ T lymphocytes *in vivo*. J Exp Med 194: 1339–1348 11696598 10.1084/jem.194.9.1339 PMC2195972

[bib32] Montaldo PG, Carbone R, Corrias MV, Ferraris PC, Ponzoni M (1994) Synergistic differentiation-promoting activity of interferon gamma and tumor necrosis factor-alfa: role of receptor regulation on human neuroblasts. J Natl Cancer Inst 22: 1694–1701 10.1093/jnci/86.22.1694 7966397

[bib33] Montaldo PG, Chiesa V, Bado M, Raffaghello L, Rozzo, Ponzoni M (1997) Induction of differentiation and apoptosis by interferon-*γ* in human neuroblastoma cells *in vitro* as a dual and alternative early biological response. Cell Death Differ 4: 150–158 16465221 10.1038/sj.cdd.4400215

[bib34] Moro M, Gasparri AM, Pagano S, Bellone M, Tornaghi P, Veglia F, Corti A, Casorati G, Dellabona P (1999) Induction of therapeutic T-cell immunity by tumor targeting with soluble recombinant B7-immunoglobulin costimulatory molecules. Cancer Res 59: 2650–2656 10363988

[bib35] Mujoo K, Cheresh DA, Yang HM, Reisfeld RA (1987) Disialoganglioside GD2 on human neuroblastoma cells: target antigen for monoclonal antibody-mediated cytolysis and suppression of tumor growth. Cancer Res 47: 1098–1104 3100030

[bib36] Salih HR, Kosowski SG, Haluska VF, Starling GC, Loo DT, Lee F, Aruffo AA, Trail PA, Kiener PA (2000) Constitutive expression of functional 4-1BB (CD137) ligand on carcinoma cells. J Immunol 165: 2903–2910 10946324 10.4049/jimmunol.165.5.2903

[bib37] Szocinski JL, Khaled AR, Hixon J, Halverson D, Funakoshi S, Fanslow WC, Boyd A, Taub DD, Durum SK, Siegall CB, Longo DL, Murphy WJ (2002) Activation-induced cell death of aggressive histology lymphomas by CD40 stimulation: induction of Bax. Blood 1: 217–223 10.1182/blood.v100.1.217 12070030

[bib38] Teitz T, Lahti JM, Kidd VJ (2001) Aggressive childhood neuroblastomas do not express caspase-8: an important component of programmed cell death. J Mol Med 79: 428–436 11511973 10.1007/s001090100233

[bib39] Teitz T, Wei T, Valentine MB, Vanin EF, Grenet J, Valentine VA, Behm FG, Look AT, Lahti JM, Kidd VJ (2000) Caspase 8 is deleted or silenced preferentially in childhood neuroblastomas with amplification of MYCN. Nat Med 6: 529–535 10802708 10.1038/75007

[bib40] Thiele CJ (1999) Neuroblastoma. In Human Cell Culture Vol. 1, Master JRW, Palsson B (eds) pp 21–53. London: Kluwer Acad Publishers

[bib41] Trinchieri G (1998) Interleukin-12: a cytokine at the interface of inflammation and immunity. Adv Immunol 70: 83–243 9755338 10.1016/s0065-2776(08)60387-9

[bib42] Turner JG, Rakhmilevich AL, Burdelya L, Neal Z, Imboden M, Sondel PM, Yu H (2001) Anti-CD40 antibody induces antitumor and antimetastatic effects: the role of NK cells. J Immunol 166: 89–94 11123280 10.4049/jimmunol.166.1.89

[bib43] Uçar K, Seeger RC, Challita PM, Watanabe CT, Yen TL, Morgan JP, Amado R, Chou E, McCallister T, Barber JR, Jolly DJ, Reynolds CP, Gangavalli R, Rosenblatt JD (1995) Sustained cytokine production and immunophenotypic changes in human neuroblastoma cell lines transduced with a human gamma interferon vector. Cancer Gene Ther 2: 171–181 8528960

[bib44] Vonderheide RH, Butler MO, Liu JF, Battle TE, Hirano N, Gribben J, Frank DA, Schultze JL, Nadler LM (2001a) CD40 activation of carcinoma cells increases expression of adhesion and major histocompatibility molecules but fails to induce either CD80/CD86 expression or T cell alloreactivity. Int J Oncol 19: 791–798 11562757 10.3892/ijo.19.4.791

[bib45] Vonderheide RH, Dutcher JP, Anderson JE, Eckhardt SG, Stephans KF, Razvillas B, Garl S, Butine MD, Perry VP, Armitage RJ, Ghalie R, Caron DA, Gribben JG (2001b) Phase I study of recombinant human CD40 ligand in cancer patients. J Clin Oncol 19: 3280–3287 11432896 10.1200/JCO.2001.19.13.3280

[bib46] Wagner HA, Gebauer M, Pollok-Kopp B, Hecker M (2002) Cytokine inducible CD40 expression in human endothelial cells is mediated by interferon regulatory factor-1. Blood 99: 520–525 11781233 10.1182/blood.v99.2.520

[bib47] Wang S, Zhu G, Chapoval AI, Dong H, Tamada K, Ni J, Chen L (2000) Costimulation of T cells by B7H2, a B7-like molecule that binds ICOS. Blood 96: 2807–2813 11023515

[bib48] Weinberg AD, Rivera MM, Prell R, Morris A, Ramstad T, Vetto JT, Urba WJ, Alvord G, Bunce C, Shields J (2000) Engagement of the OX-40 receptor *in vivo* enhances antitumor immunity. J Immunol 164: 2160–2169 10657670 10.4049/jimmunol.164.4.2160

[bib49] Wilson JL, Charo J, Martin-Fonteche A, Dellabona P, Casorati G, Chambers BJ, Kiessling R, Bejarano MT, Ljunggren HG (1999) NK cell triggering by the human costimulatory molecules CD80 and CD86. J Immunol 163: 4207–4712 10510357

[bib50] Wu TC, Huang AY, Jaffee EM, Levitsky HI, Pardoll DM (1995) A reassessment of the role of B7-1 expression in tumor rejection. J Exp Med 182: 1415–1421 7595212 10.1084/jem.182.5.1415 PMC2192183

